# Glutathione contributes to plant defence against parasitic cyst nematodes

**DOI:** 10.1111/mpp.13210

**Published:** 2022-03-29

**Authors:** M. Shamim Hasan, Divykriti Chopra, Anika Damm, Anna Koprivova, Stanislav Kopriva, Andreas J. Meyer, Stefanie Müller‐Schüssele, Florian M. W. Grundler, Shahid Siddique

**Affiliations:** ^1^ Institute of Crop Science and Resource Conservation (INRES) Molecular Phytomedicine University of Bonn INRES Bonn Germany; ^2^ Department of Plant Pathology Faculty of Agriculture Hajee Mohammad Danesh Science and Technology University Dinajpur Bangladesh; ^3^ 14309 Institute for Plant Sciences Cluster of Excellence on Plant Sciences University of Cologne Cologne Germany; ^4^ 9374 Institute of Crop Science and Resource Conservation (INRES) Chemical Signalling University of Bonn Bonn Germany; ^5^ Department of Entomology and Nematology University of California Davis California USA

**Keywords:** glutathione, nematode, plant‐parasitic nematode, redox, syncytium

## Abstract

Cyst nematodes (CNs) are an important group of root‐infecting sedentary endoparasites that severely damage many crop plants worldwide. An infective CN juvenile enters the host's roots and migrates towards the vascular cylinder, where it induces the formation of syncytial feeding cells, which nourish the CN throughout its parasitic stages. Here, we examined the role of glutathione (l‐γ‐glutamyl‐l‐cysteinyl‐glycine) in *Arabidopsis thaliana* on infection with the CN *Heterodera schachtii*. *Arabidopsis* lines with mutations *pad2*, *cad2*, or *zir1* in the glutamate–cysteine ligase (*GSH1*) gene, which encodes the first enzyme in the glutathione biosynthetic pathway, displayed enhanced CN susceptibility, but susceptibility was reduced for *rax1*, another *GSH1* allele. Biochemical analysis revealed differentially altered thiol levels in these mutants that was independent of nematode infection. All glutathione‐deficient mutants exhibited impaired activation of defence marker genes as well as genes for biosynthesis of the antimicrobial compound camalexin early in infection. Further analysis revealed a link between glutathione‐mediated plant resistance to CN infection and the production of camalexin on nematode infection. These results suggest that glutathione levels affect plant resistance to CN by fine‐tuning the balance between the cellular redox environment and the production of compounds related to defence against infection.

## Graphical abstract

Glutathione contributes to plant resistance against cyst nematodes by activating plant defence responses, including production of camalexin.

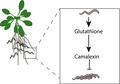

## 1 INTRODUCTION

Thiol‐containing compounds are central components of many pharmacological and biochemical reactions. In its reduced form, glutathione (l‐γ‐glutamyl‐l‐cysteinyl‐glycine, GSH) is the most abundant low‐molecular‐weight thiol in most cells. The thiol group of the central cysteine residue, which has a nucleophilic character, is required for reduction and conjugation reactions in which glutathione plays pivotal roles. Besides maintaining tight control of cellular redox status via its reducing and antioxidant properties, glutathione mediates many other physiological processes, such as cellular signalling, thiol‐disulphide interchange reactions, and xenobiotic metabolism, and serves as a major component of the cysteine pool (Noctor et al., 2012).

Glutathione biosynthesis generally occurs via two ATP‐dependent steps (Meister, 1995). In the first reaction, a peptide bond forms between the α‐amino group of l‐cysteine (l‐Cys) and the γ‐carboxyl of l‐glutamate (l‐Glu) via a process catalysed by glutamate–cysteine ligase (GSH1). In the second step, glutathione synthetase (GSH2; Hell & Bergmann, 1990) adds glycine (Gly) to the dipeptide γ‐glutamyl‐cysteine (γ‐EC) to form GSH. In *Arabidopsis thaliana*, these two enzymes are encoded by single‐copy genes; GSH1 is exclusively localized to chloroplasts, whereas GSH2 is predominantly located in the cytosol and to a lesser extent in plastids (Pasternak et al., 2008; Wachter et al., 2005). Under stress conditions, reduced glutathione is rapidly converted into the oxidized form glutathione disulphide (GSSG), leading to an imbalance in the glutathione redox potential (*E*_GSH_), which is considered an indicator of oxidative stress. The accumulation of GSSG is counteracted by glutathione reductases, which reduce GSSG back to GSH at the expense of electrons provided by NADPH (Marty et al., 2009, 2019).

Glutathione also functions as a co‐substrate in conjugation and detoxification reactions catalysed by glutathione transferases (GSTs), and in glutathionylation of protein thiols as a posttranslational protein modification (Dixon et al., 2005). In addition, glutathione acts in plant perception systems to activate basal defence responses that help counter microbial attack (Glazebrook & Ausubel, 1994; Parisy et al., 2007). In coordination with cysteine, glutathione plays a role in the establishment and signalling of pathogen‐associated molecular pattern (PAMP)‐triggered immunity (PTI) (Alvarez et al., 2011; Jones & Dangl, 2006).

GSH1 is thought to be the rate‐limiting enzyme for GSH synthesis (Arisi et al., 1997). Null mutants of *GSH1* in *Arabidopsis* are embryo‐lethal (Cairns et al., 2006). Less severe mutations within this gene appear in five distinct mutant alleles (*rml1*, *rax1*, *pad2*, *cad2*, and *zir1*) that were identified through forward genetic screens and found to show a partial decrease in glutathione production (Ball et al., 2004; Bangash et al., 2019; Cobbett et al., 1998; Glazebrook & Ausubel, 1994; Shanmugam et al., 2012; Vernoux et al., 2000). Studies of these *GSH1*‐deficient mutants have shed light on the diverse roles of glutathione in many cellular processes, including plant development and responses to abiotic and biotic stimuli (Foyer & Noctor, 2011; Noctor, 2006; Potters et al., 2002). The *Arabidopsis root‐meristemless1* (*rml1*) mutant possesses <5% of wild‐type levels of foliar glutathione and exhibits severe defects in plant development (Vernoux et al., 2000). Another *Arabidopsis GSH1* mutant, *zinc tolerance induced by iron 1* (*zir1*), contains <15% of wild‐type levels of glutathione in leaves, leading to deficits in Fe‐dependent Zn tolerance and impaired nitric oxide (NO)‐mediated Fe‐deficiency signalling (Shanmugam et al., 2012, 2015). The *cadmium‐sensitive2* (*cad2*) mutant contains c.30% of wild‐type glutathione levels and shows sensitivity to cadmium and enhanced susceptibility to *Phytophthora brassicae* (Cobbett et al., 1998; Howden et al., 1995; Parisy et al., 2007). The *regulator of ascorbate peroxidase2 1* (*rax1*) mutant contains c.40% of wild‐type levels of glutathione in leaves and shows increased sensitivity to light stress (Ball et al., 2004). However, the altered glutathione content in *rax1* does not appear to affect plant resistance to *Pseudomonas syringae* or *P*. *brassicae* (Parisy et al., 2007). The *phytoalexin deficient2* (*pad2*) mutant, the most extensively studied mutant in an allelic series of *GSH1* mutants, possesses only c.20% of wild‐type levels of glutathione in leaves and shows enhanced susceptibility to many pests and pathogens (Dubreuil‐Maurizi & Poinssot, 2012). Despite substantial evidence for the role of glutathione in different plant pathosystems, little is known about how glutathione mediates the molecular dialogue during plant–nematode interactions.

Plant‐parasitic cyst nematodes (CNs) are among the most damaging plant pests and pathogens, causing substantial yield losses globally (Savary et al., 2019). Infective‐stage juveniles (J2) of CNs hatch from eggs on stimulation by mostly unknown host triggers and migrate toward the roots. The CNs make numerous perforations in the epidermal cell wall via back‐and‐forth movements with their stylets and invade the roots of the host plant near the root tip. Subsequently, they migrate intracellularly through the root cortical cells to the vascular cylinder. On reaching the vascular cylinder, the nematodes probe single cambial or procambial cells to induce the formation of an initial syncytial cell (ISC) as a feeding site (Sobczak et al., 1999). The nematodes inject a cocktail of secretions into the ISC through their hollow stylets to modify plant morphogenetic pathways towards the development of the feeding site (Hewezi & Baum, 2013; Wyss & Zunke, 1986). Hundreds of adjacent root cells successively fuse with the ISC via local cell wall dissolution to form a hypertrophied, multinucleate, hypermetabolic syncytial nurse cell (Wyss & Grundler, 1992).

The juvenile nematode rapidly becomes sedentary due to cell‐specific muscle atrophy and starts feeding on the syncytium, which acts as a nutrient sink throughout its parasitic stages (Han et al., 2018). Syncytium development is accompanied by extensive metabolic, transcriptomic, and proteomic changes in the infected root tissues (Hofmann et al., 2010; Hütten et al., 2015; Siddique et al., 2009; Szakasits et al., 2009). The juveniles feed, enlarge, and moult three times to differentiate into males or females. Female nematodes grow rapidly and burst through the root surface, while males regain a vermiform body shape and mobile form by remodelling their neuromuscular structures, leave the roots, and search for females (Han et al., 2018). The female dies after fertilization, and the body wall tans to form a typical brown cyst that envelops and shields the next generation of eggs. The eggs are able to survive for prolonged periods (up to 20 years) in the soil until a suitable host is found growing nearby (Grainger, 1964).

The beet CN (*Heterodera schachtii*) is a detrimental pest of sugar beet worldwide. *H*. *schachtii* infects over 200 plants from 20 different families, including the model plant *Arabidopsis* (Sijmons et al., 1991). Here, using the *Arabidopsis* and *H*. *schachtii* model system, we investigated the role of glutathione in orchestrating host defence responses to CN infection. We conclude that glutathione contributes to plant defence against CN infection through modulation of cellular redox homeostasis and camalexin production in host roots.

## 2 RESULTS

### 2.1 Cyst nematode infection activates GSH biosynthesis genes in *Arabidopsis*

To investigate the roles of glutathione biosynthetic genes during different phases of *Arabidopsis* parasitism by CN, we examined the expression patterns of *GSH1* and *GSH2* in publicly available transcriptomic data (Mendy et al., 2017; Siddique et al., 2021; Szakasits et al., 2009). These surveys revealed a significant increase in *GSH1* expression during the migratory (10 h postinoculation, hpi) and sedentary (5 and 15 days postinfection, dpi) stages of cyst nematode infection (Table S1 and Figure S1). In comparison, although *GSH2* transcript abundance increased significantly during the migratory stage, it remained unchanged during the sedentary stage of infection.

**FIGURE 1:**
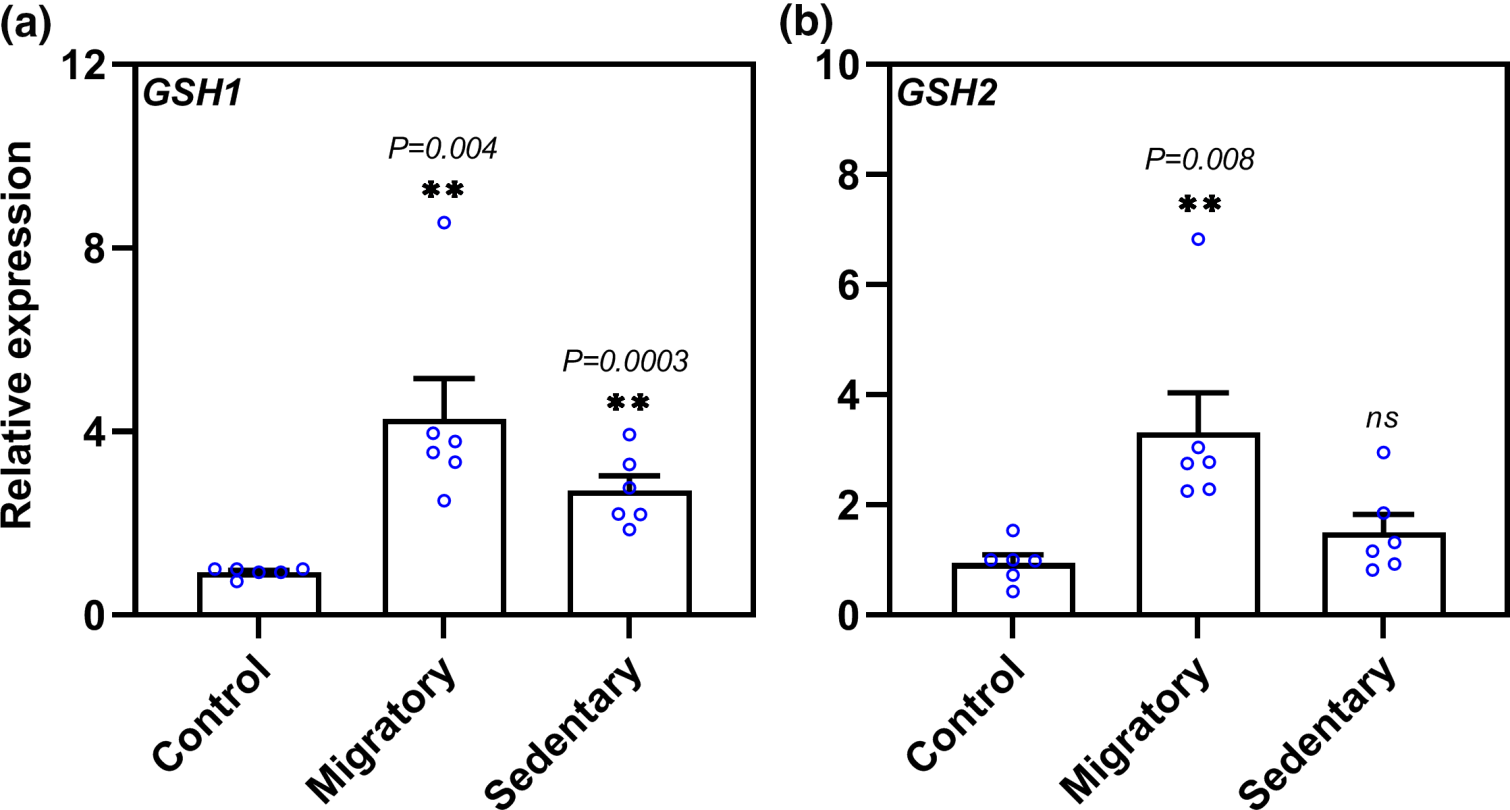
Glutathione (GSH) biosynthesis genes are induced by infection with *Heterodera schachtii*. Validation of changes in expression of genes coding for glutathione biosynthesis enzymes GSH1 (a) and GSH2 (b) during the migratory and sedentary stages of nematode infection via reverse transcription‐quantitative PCR (RT‐qPCR). Values represent relative expression levels on CN infection, with the value of the first replicate in uninfected control roots (Col‐0) set to 1. The mRNA levels were measured in three technical replicates per sample. The transcript level of each gene was normalized to that of the *Arabidopsis* housekeeping gene *18S rRNA* and *UBQ10* with three biological replicates each (blue circle). Data are presented as the mean ± *SE*. Each biological replicate contained a pool of hundreds of small root segments with infection sites at 10 h postinoculation (hpi) (‘Migratory’) or syncytia at 10 days postinoculation (dpi) (‘Sedentary’). Data were analysed with a two‐tailed Student's *t* test (*p* < .05) and asterisks represent significant differences compared to the uninfected control root

To validate these microarray data, we analysed the expression of GSH biosynthesis genes in *Arabidopsis* root segments infected with CN. We collected several hundred root segments (c.0.2 cm) containing infection sites at 10 hpi (migratory stage) and syncytia at 10 dpi (sedentary stage), and analysed *GSH1* and *GSH2* expression in these tissues compared with uninfected wild‐type Columbia (Col‐0) roots by reverse transcription‐quantitative PCR (RT‐qPCR). The results confirmed that *GSH1* transcript levels increased during both the migratory and sedentary stages of CN infection (Figure 1a), whereas *GSH2* was significantly induced at 10 hpi but not at 10 dpi (Figure 1b). Overall, these results suggest that GSH biosynthesis genes are activated on CN infection, and that the regulation of *GSH1* is more pronounced than that of *GSH2* during CN infection; therefore, we focused our further analysis on *GSH1*.

### 2.2 Altered glutathione levels in plants influence cyst nematode infection and development

**FIGURE 2:**
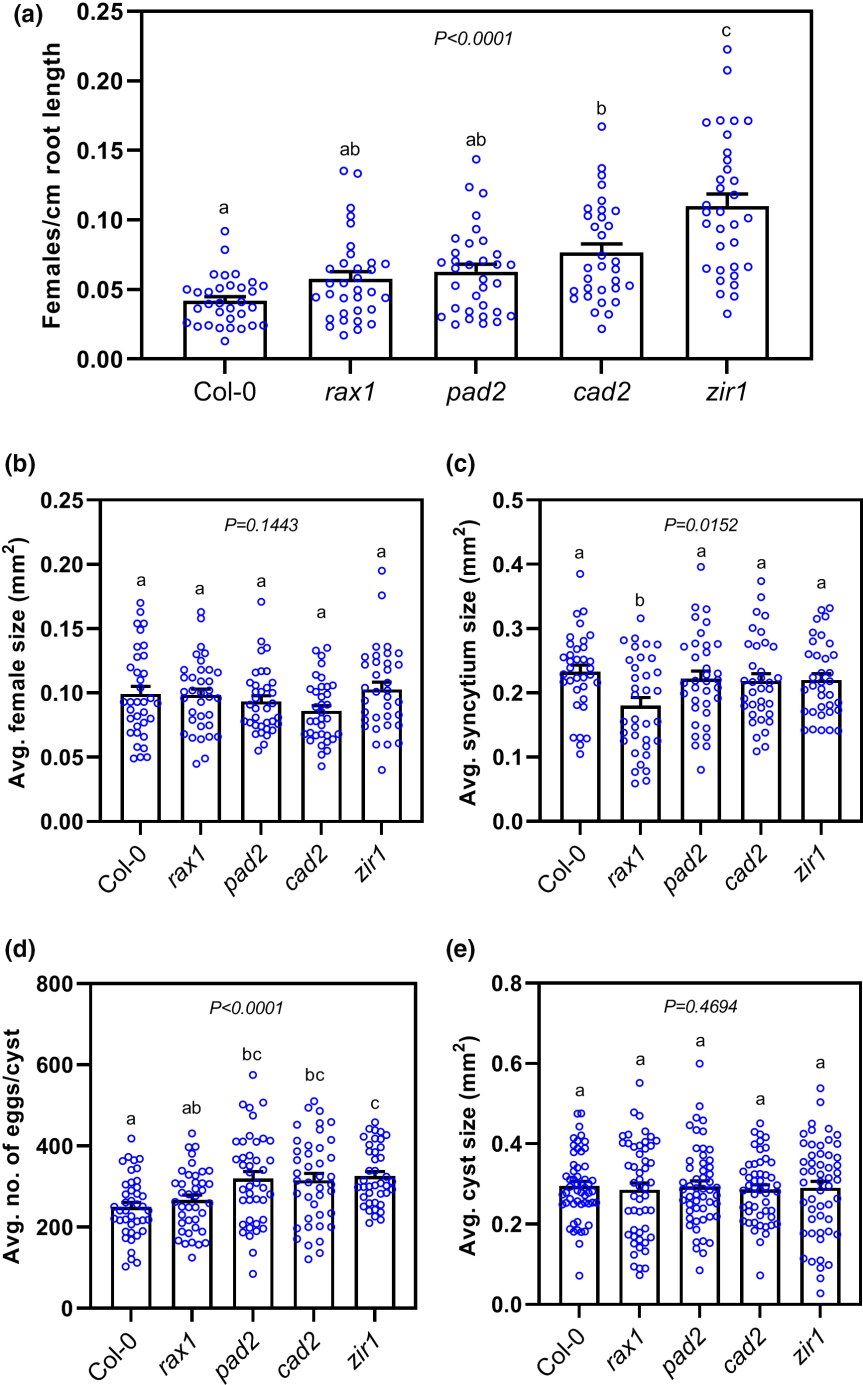
Effect of glutathione (GSH) biosynthesis mutations on the susceptibility of *Arabidopsis* to *Heterodera schachtii*. (a) Nematode infection assay showing the number of females/cm root length of *GSH1* mutant lines versus wild‐type Col‐0. Approximately 60–70 *H*. *schachtii* infective juveniles were inoculated onto 12‐day‐old *Arabidopsis* plants. The number of females per root system was counted at 14 days postinoculation (dpi), and the infection rate per centimetre of root length was determined after scanning the roots using the WinRhizo root image analysis system. (b) Average size of female nematodes at 14 dpi. Approximately 30–40 female nematodes were randomly selected and their outlines measured for each biological replicate. (c) Average size of syncytia at 14 dpi. Approximately 30–40 female‐associated syncytia in each replicate were measured using a stereomicroscope. (d) Average number of eggs per cyst at 42 dpi. For each biological replicate, 40–45 cysts were randomly selected and crushed between slides, their contents transferred to a counting dish, and the number of J2s/eggs counted under a stereomicroscope. (e) Average cyst size (50–55 per repetition) at 42 dpi. Bars represent mean ± *SE*. All data were analysed using one‐way analysis of variance followed by Tukey's HSD post hoc test. Different letters indicate significantly different means (95% confidence). Data represent one of three independent experiments with similar results

To assess whether glutathione levels in *Arabidopsis* affect CN parasitism, we screened an allelic series of *GSH1* and mutants in nematode infection assays by measuring multiple nematode susceptibility parameters. We grew plants under sterile conditions in agar medium, and when the roots spread through the agar we inoculated them with 60–70 J2 nematodes per plant. At 14 dpi, we counted the average number of females, a widely accepted parameter for nematode susceptibility under in vitro conditions. The average number of females per centimetre of root length was significantly higher in *zir1* and *cad2* than in the Col‐0 control (Figure 2a). However, *rax1* and *pad2* plants showed no difference in the number of females as compared to Col‐0 (Figure 2a). Next, we measured the size of female nematodes and female‐associated syncytia at 14 dpi. There was no significant difference in either parameter in any lines carrying allelic mutations in *GSH1*, except for *rax1*, which, surprisingly, displayed a slight decrease in the size of female‐associated syncytia relative to the wild type (Figure 2b,c). Finally, we compared cyst egg contents and cyst size at 42 dpi. We detected substantially more eggs per cyst in *pad2*, *cad2*, and *zir1* than Col‐0, but not in *rax1* plants (Figure 2d). However, the cyst size was unaffected in all *gsh1* mutants examined (Figure 2e).

### 2.3 Changes in the susceptibility of glutathione‐deficient mutants are unrelated to CN attraction

To elucidate whether the changes in the susceptibility of the *GSH1* mutants to *H*. *schachtii* were associated with the attractiveness of the plant roots, we performed nematode attraction assays using agar discs containing root exudates from 12‐day‐old mutants and wild‐type control plants. Agar discs containing root exudates of Col‐0 plants attracted more J2s than control agar discs (without roots), indicating that nematodes were able to sense the signal from the root exudates (Figure S2). However, we observed no significant difference in attractiveness towards the root exudates for any *GSH1* mutant compared with control plants (Figure S2). Thus, the changes in the glutathione‐deficient mutants’ susceptibility to CN infection are probably not associated with any factor(s) mediating the plants’ attractiveness to nematodes.

### 2.4 Thiol levels are altered in *GSH1* mutants, regardless of CN infection

**FIGURE 3:**
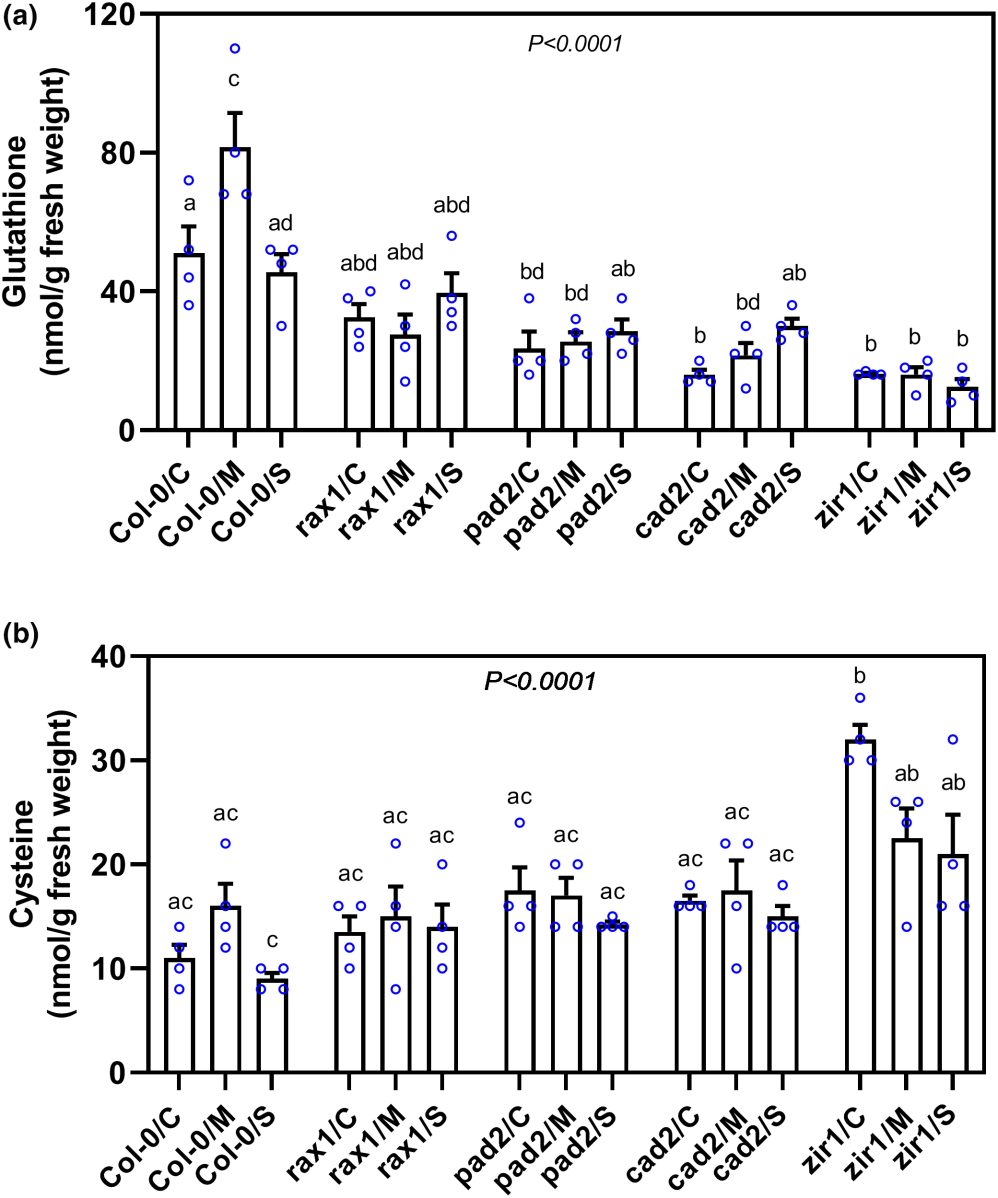
Thiol contents in uninfected roots and cyst nematode infection sites in the *GSH1* mutants *rax1*, *pad2*, *cad2*, and *zir1*. Several hundred small root segments (c.0.2 cm) containing infection sites at the migratory (10 h postinoculation [hpi]) and sedentary (10 days postinoculation [dpi]) stages were collected for measurement of glutathione (a) and cysteine (b) contents by high‐performance liquid chromatography (HPLC). Data points represent four independent experiments (means ± *SE*). Different lowercase letters denote significant differences, as determined by analysis of variance (*p* < .05) followed by Tukey's HSD post hoc tests. C, control; M, migratory; S, sedentary

*Arabidopsis* plants harbouring mutant alleles of *GSH1* contain constitutively reduced foliar glutathione levels (Parisy et al., 2007; Shanmugam et al., 2012). We explored the extent to which glutathione accumulation is affected in roots due to mutations in *GSH1*. For this purpose, we collected uninfected root tissues from 12‐day‐old plants and measured glutathione levels. We observed a similar trend in root glutathione levels in *GSH1* mutants as in the foliar levels reported previously (Parisy et al., 2007; Shanmugam et al., 2012), but these levels differed: *rax1* had the highest glutathione level (54% of Col‐0), followed by *pad2* (46%), *cad2* (31%), and *zir1* (30%; Figure 3a). Next, we measured glutathione levels in root pieces with infection sites at 10 hpi (migratory stage) and 10 dpi (sedentary stage). Glutathione levels were significantly higher in infected Col‐0 at 10 hpi than in uninfected control roots at 10 hpi. However, glutathione returned to uninfected control levels at 10 dpi. In contrast to Col‐0, no increase was detected in glutathione levels in the roots of any of the *GSH1* mutant on nematode infection (Figure 3a).

Cysteine functions as a metabolic precursor for numerous essential biomolecules, such as glutathione, glucosinolates, and phytoalexins (Meyer & Hell, 2005; Rausch & Wachter, 2005). Because the amount of cysteine often inversely correlates with the amount of GSH (Speiser et al., 2018), we measured cysteine levels in roots during different stages of CN infection. Surprisingly, the uninfected roots of *GSH1* mutants contained wild‐type levels of cysteine except for *zir1*, which showed a significant increase in cysteine compared with Col‐0 (Figure 3b). Similarly, we observed no notable changes in cysteine accumulation in infected root segments of mutant plants at 10 hpi or 10 dpi compared with Col‐0 (Figure 3b).

### 2.5 Glutathione‐depleted mutants have impaired basal defence to CN

**FIGURE 4:**
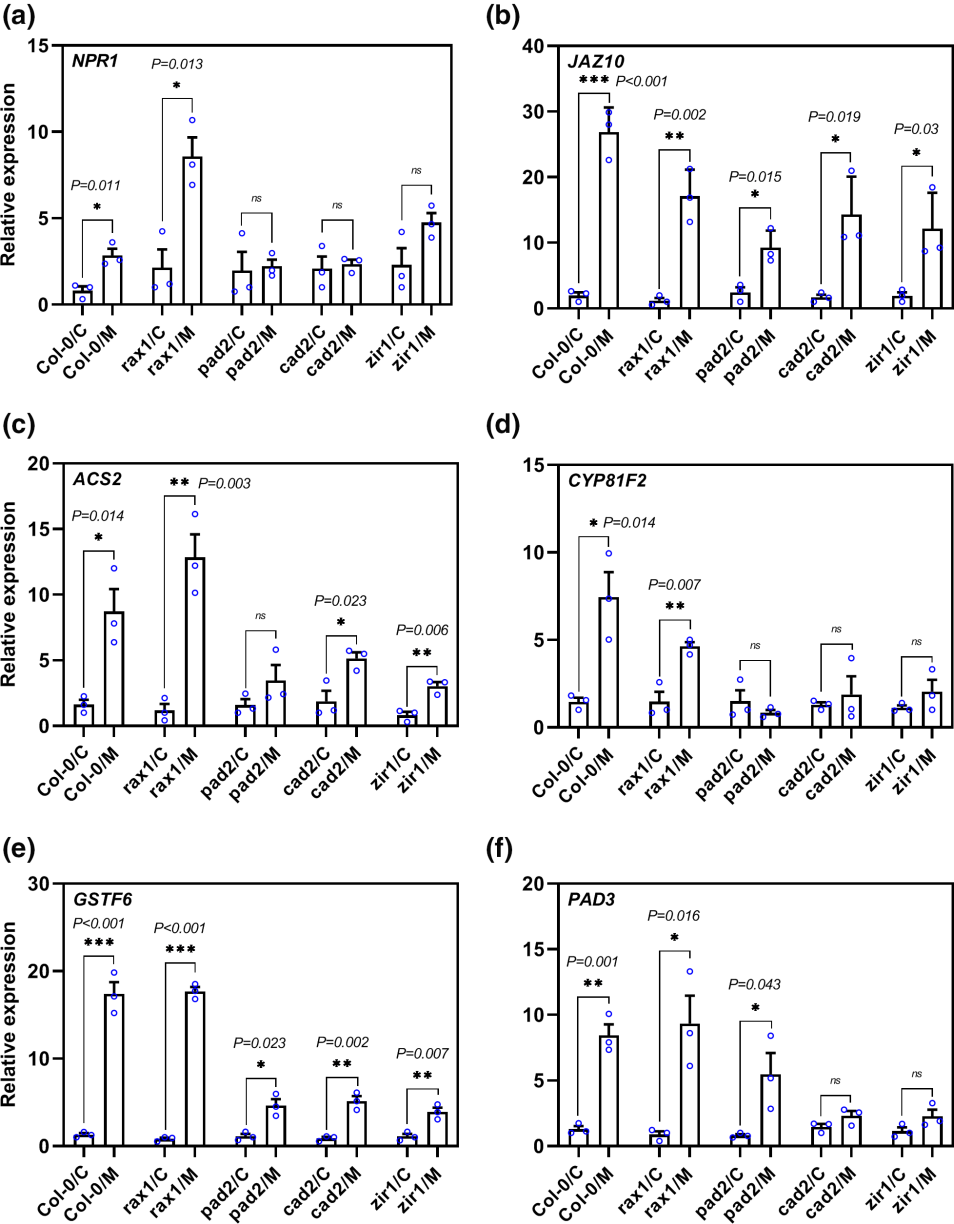
Reduced glutathione levels lead to impaired basal defence responses. (a–f) Expression of defence marker genes in response to *Heterodera schachtii* infection during the migratory stage in roots of glutathione (GSH)‐deficient mutants. Several hundred root segments (c.0.2 cm) containing infection sites were collected at 10 h postinoculation (hpi) and the expression levels of *NPR1*, *JAZ10*, *ACS2*, *CYP81F2*, *GSTF6*, and *PAD3* were analysed by reverse transcription‐quantitative PCR (RT‐qPCR). Expression levels of the genes of interest were normalized to the *Arabidopsis* housekeeping gene *18S rRNA*. Data represent relative expression levels of the indicated genes, with the value for uninfected roots of each genotype set to 1. Asterisks represent significant difference in expression levels from the corresponding uninfected control roots. Data represent means ± *SE* (*n* = 3 biological replicates, *p < .05, **p < .01, ***p < .001, using two‐tailed Student's *t* test). C, control; M, migratory stage of infection; ns, nonsignificant

We hypothesized that the hypersusceptibility of GSH‐deficient mutants might be due to impaired expression of genes in defence‐related pathways. Therefore, we assessed the transcript abundance of following six plant basal defence marker genes that are strongly up‐regulated during the migratory stage of infection (Mendy et al., 2017; Shah et al., 2017): *NPR1*, a salicylic acid signalling gene (Yan & Dong, 2014); *ACS2*, which is involved in ethylene signalling (Yamagami et al., 2003); *JAZ10*, a jasmonic acid signalling gene (Chini et al., 2007); and three genes associated with indole‐glucosinolate and camalexin biosynthesis pathways, namely, the genes encoding GSTF6 (mediates the conjugation between indole‐3‐acetonitrile [IAN] and GSH; Su et al., 2011), CYP71B15 (PAD3; catalyses the conversion of dihydrocamalexic acid [DHCA] to its final form [camalexin] in the camalexin biosynthesis pathway; Schuhegger et al., 2006), and CYP81F2 (involved in the catabolism of indol‐3‐yl‐methyl glucosinolate; Clay et al., 2009). The results from RT‐qPCR showed no significant differences in the expression levels of any of the tested marker genes between wild‐type uninfected roots and those of the *GSH1* mutants (Figure S3). Next, we sampled small root segments (c.0.2 cm) containing infection sites at the migratory stage of CN infection (10 hpi) and analysed gene expression by RT‐qPCR. As previously observed (Mendy et al., 2017; Shah et al., 2017), expression of all tested genes was significantly increased in Col‐0 on infection compared with uninfected control roots (Figure 4a–f). In comparison to Col‐0, normal increase in transcript levels of all tested genes was impaired in *pad2*, *cad2*, and *zir1* (Figure 4a–f). Interestingly, *rax1*showed a normal or even more pronounced increase in defence marker gene expression on infection (Figure 4a–f). Together, these findings suggest that glutathione is involved in activation of plant defence responses, including indole‐glucosinolate and camalexin biosynthesis pathways, on CN infection in *Arabidopsis* roots.

### 2.6 CN parasitism causes shifts in the redox state of glutathione

**FIGURE 5:**
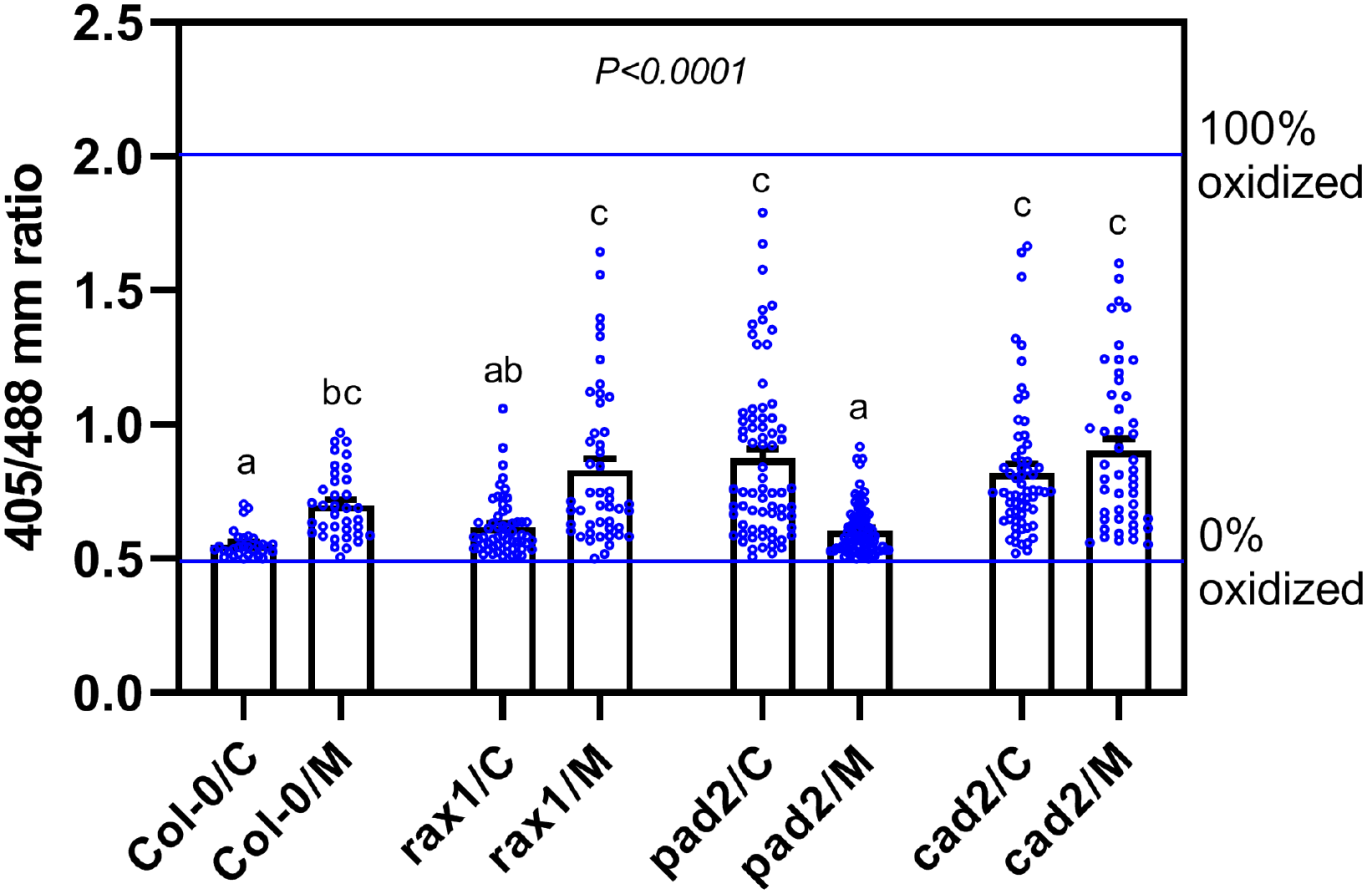
Response of the glutathione redox potential in the cytosol of uninfected and infected (10 h postinoculation [hpi]) root cells measured with Grx1‐roGFP2. The fluorescent probe Grx1‐roGFP2 was subsequently excited at 405 and 488 nm, and fluorescence recorded at 505–530 nm. For calibration (determining 0% oxidation and 100% oxidation of Grx1‐roGFP2), 5 mM 2,2′‐dipyridyldisulfide (DPS) solution was used as an oxidizing agent and 10 mM dithiothreitol (DTT) solution was used as a reducing agent. The respective ratios are depicted as horizontal lines in the diagram. Data points denote individual ratio measurements from regions of interest near infection sites. Results are from three biological replicates (bar chart depicts means ± *SE*). Different lowercase letters denote significant differences, as determined by one‐way analysis of variance (*p* < .05) followed by Tukey's HSD post hoc tests. C, control; M, migratory

The glutathione‐dependent fluorescent probe Grx1‐roGFP2 is commonly used to monitor *E*_GSH_ at the subcellular level in vivo (Meyer et al., 2007; Müller‐Schüssle et al., 2021; Schwarzländer et al., 2008). To assess whether CN parasitism causes changes in *E*_GSH_, we performed ratiometric analysis of Grx1‐roGFP2 (Gutscher et al., 2008; Wagner et al., 2019) expressed in the cytosol via confocal microscopy following excitation at 405 and 488 nm. We grew plants in 35‐mm Petri dishes with a 14‐mm glass microwell and inoculated them with 50 J2s of CNs. For calibration, we exposed uninfected roots to reducing (10 mM dithiothreitol [DTT]) and oxidizing (5 mM 2,2′‐dipyridyldisulfide [DPS]) reagents. We found that the 405/488 nm fluorescence ratio in Col‐0 roots was slightly increased at 10 hpi, indicating a less negative *E*_GSH_ (Figure 5a). Together with the increase in total GSH during the migratory state (Figure 3a) this result suggests a slight oxidation of the glutathione pool (Figure 5a). A similar trend albeit with larger variance towards an even more pronounced oxidation was found for *rax1* (Figure 5a). In both *pad2* and *cad2* under noninfected control conditions, an increased ratio indicated a less reducing *E*_GSH_, which was expected from the pronounced decrease in total glutathione (Meyer et al., 2007). While *cad2*, which also displayed enhanced susceptibility to *H*. *schachtii*, showed no change in Grx1‐roGFP2 oxidation on infection, *pad2* surprisingly showed a more reducing *E*_GSH_ after infection (Figure 5a).

### 2.7 GSH‐mediated camalexin levels in roots determine plant susceptibility to CN infection

**FIGURE 6:**
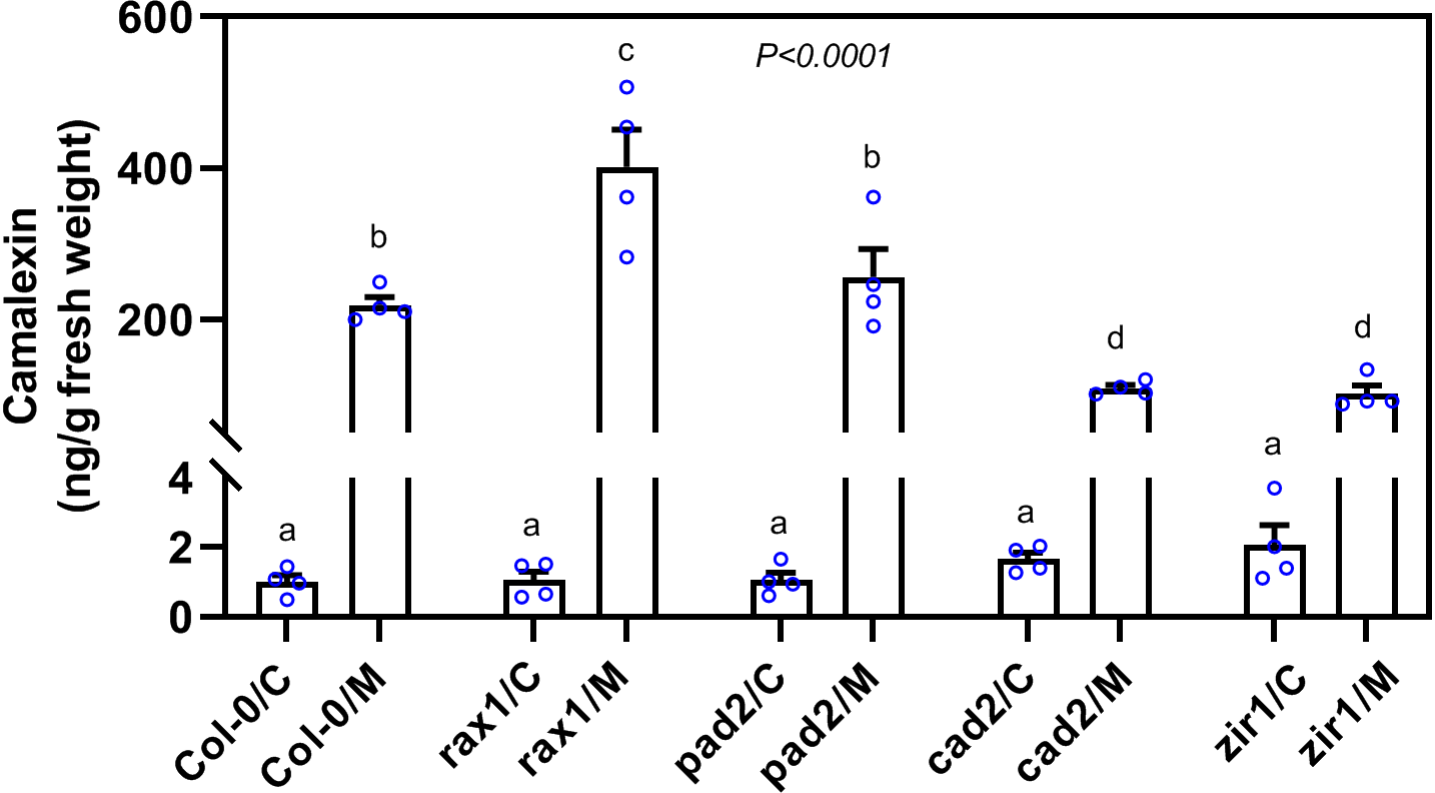
Camalexin levels in uninfected and cyst nematode‐infected roots during the migratory stage (10 h postinoculation [hpi]) in glutathione (GSH)‐deficient mutants measured by high‐performance liquid chromatography (HPLC). Data points represent four independent experiments (means ± *SE)*. Data were analysed by one‐way analysis of variance followed by Tukey's HSD post hoc multiple comparisons test (*p* < .0001). Identical lowercase letters denote no significant differences. C, control; M, migratory stage of infection

To explore whether there is a link between glutathione and nematode‐induced camalexin biosynthesis, we used high‐performance liquid chromatography (HPLC) to measure camalexin levels in root segments at 10 hpi and in uninfected roots. In uninfected roots, camalexin levels were low and no significant differences in camalexin content were detected between Col‐0 and glutathione‐deficient mutants. However, we observed an almost 200‐fold increase in camalexin content on CN infection in Col‐0 (Figure 6). Interestingly, an even more pronounced increase was found in the camalexin level in *rax1* roots on CN infection (Figure 6). However, in both *cad2* and *zir1* roots, the increase in camalexin levels was significantly lower than for Col‐0 following CN infection, and in *pad2* roots induction was the same (Figure 6).

## 3 DISCUSSION

In the present work, we use *Arabidopsis* GSH biosynthesis mutants to examine how glutathione contributes to plant susceptibility to CN infection. We determined that the expression of *GSH1* and *GSH2* is significantly increased in response to CN infection during the migratory stage of CN infection. CNs cause extensive damage to host root tissues during their migration inside the roots (Shah et al., 2017). In turn, reactive oxygen species (ROS) production and the detoxification machinery are simultaneously switched on to fine‐tune the activation of a network of plant defence responses (Ithal et al., 2007; Mazarei et al., 2011; Molinari & Miacola, 1997; Siddique et al., 2014). Increased expression of *GSH1* and *GSH2* therefore may be a response to the high demand for glutathione to re‐establish ROS homeostasis after an infection or for synthesis of the sulphur‐containing phytoalexin camalexin during the migratory stage of infection (Parisy et al., 2007; Siddique et al., 2014).

While null mutants of *GSH1* in *Arabidopsis* are embryo‐lethal (Cairns et al., 2006), five distinct mutant alleles (*rml1*, *rax1*, *pad2*, *cad2*, and *zir1*) show a partial decrease in glutathione production (Ball et al., 2004; Bangash et al., 2019; Cobbett et al., 1998; Glazebrook & Ausubel, 1994; Shanmugam et al., 2012; Vernoux et al., 2000). Of these, *rml1* contained <5% of the wild‐type levels of glutathione and showed aborted root growth (Vernoux et al., 2000). Because this severe phenotype compromised the analysis of *rml1*, we analysed plant–CN interactions in the four other *GSH1* mutants (*rax1*, *pad2*, *cad2*, and *zir1*). We found that glutathione content increased significantly during the migratory stage of infection in Col‐0 compared with uninfected control plants, which supports our observation of increased GSH biosynthetic gene expression. However, glutathione levels were consistently low in *GSH1* mutant plants (*rax1, pad2*, *cad2*, and *zir1*) with and without infection. Notably, three out of four *gsh1* mutants (*pad2*, *cad2*, and *zir1*) also showed enhanced susceptibility to CN, suggesting a positive role for glutathione in host defence activation on CN infection.

Cellular redox homeostasis relies on the equilibrium between the oxidized and reduced forms of glutathione and influences many cellular processes via direct or indirect regulation at the gene or protein level (Ball et al., 2004; Cobbett et al., 1998; Jez et al., 2004; Mou et al., 2003). The increased glutathione accumulation in response to infection suggests that the hypersusceptibility of the glutathione mutants to CN might be associated with disturbances in the cellular redox status of the host plant. Monitoring the fluorescent *E*_GSH_ sensor Grx1‐roGFP2 in vivo, we found a more oxidized probe during the migratory stage of nematode infection in Col‐0. Interestingly, *rax1* mutants showed an oxidative shift of *E*_GSH_, as Col‐0, and partially retained induction of defence gene expression. In contrast, this oxidative *E*_GSH_ shift at 10 hpi was absent in the more susceptible *cad2* and *pad2* plants. This finding suggests that glutathione deficiency interferes with pathogen‐triggered signalling events that are crucial for successful nematode parasitism, leading to increased plant susceptibility to CN.

The importance of GSH for camalexin accumulation and for disease resistance has been previously demonstrated (Glazebrook & Ausubel, 1994; Parisy et al., 2007). Furthermore, loss‐of‐function camalexin biosynthesis mutants have been shown to display enhanced susceptibility to CN (Ali et al., 2014; Shah et al., 2017). Here, we found that the up‐regulation of two key camalexin biosynthesis genes in response to CN infection was impaired in GSH‐deficient mutants as compared with Col‐0 control plants. Furthermore, camalexin accumulation in response to CN infection was significantly lower in the roots of *cad2* and *zir1* plants. Based on these data, we propose that an adequate level of GSH is required for camalexin accumulation, which in turn plays a role in the plant's defence against CN infection.

Surprisingly, the *rax1* mutant showed a different biochemical phenotype than the other *GSH1* mutants, with significantly higher levels of camalexin during early nematode parasitism. Moreover, the cellular redox state was more oxidized in *rax1* than in the other mutants, pointing to oxidative stress conditions, which might ultimately influence camalexin accumulation in these plants. In our experiments, *rax1* was noted to contain 54% of the wild‐type level of glutathione, the highest amount produced by any *GSH1* mutant, indicating that it is able to utilize cysteine to maintain glutathione biosynthesis at a level sufficient to resist nematode invasion. Indeed, *rax1* showed a significant reduction in the size of female‐associated syncytia. Moreover, *rax1* shows increased *ASCORBATE PEROXIDASE2* (*APX2*) expression in response to wounding (Ball et al., 2004). Loss‐of‐function *apx2* mutants are compromised in ROS production during oxidative stress (Suzuki et al., 2013). *APX2* expression is probably triggered by *H*. *schachtii* during its destructive invasion and migration, leading to excessive ROS accumulation in the roots of *rax1* plants. Although the *rax1* mutant displays lower ROS accumulation under nonstress conditions, and CNs can utilize ROS for successful parasitism, the mutant's overproduction of ROS on nematode infection (caused by the misregulation of the ascorbate–glutathione cycle, resulting in oxidative stress and enhanced camalexin production) might hamper nematode development (Ball et al., 2004; Noctor & Foyer, 1998; Siddique et al., 2014; Tierens et al., 2002; Zhao et al., 1998).

In summary, our findings suggest that the precise regulation of glutathione homeostasis is crucial for mounting an effective plant defence response to CN infection. We propose that glutathione depletion is positively correlated with camalexin accumulation in roots on CN infection. Accumulation of camalexin in turn perturbs the cellular redox state and activates signalling events that negatively influence plant susceptibility to CNs.

## 4 EXPERIMENTAL PROCEDURES

### 4.1 Plant material and growth conditions

*A. thaliana* seeds were disinfected by washing in 2% sodium hypochlorite (wt/vol) for 3 min, followed by washing with 70% (vol/vol) ethanol for 5 min and rinsing three times consecutively with sterile water. After being dried on sterile Whatman filter paper for 2–4 h, seeds were stored at 4°C before plating. Sterilized seeds were sown in Petri dishes with agar medium enriched with modified Knop's nutrient solutions as previously described (Sijmons et al., 1991). Plants were grown under long‐day conditions with 16 h of light and 8 h of darkness in a growth chamber at 23°C for CN infection (Siddique et al., 2015).

### 4.2 Validation of mutant lines

The mutant lines *pad2*, *cad2*, *rax1*, and *zir1* used in our study were validated by PCR. The area surrounding the predicted point mutation was amplified by PCR using the primers listed in Table S2 and subsequently the single point mutation/deletion was verified by sequencing. The genotyping results of the mutants are presented in Figure S4.

### 4.3 Nematode infection assay

*H. schachtii* cysts were harvested from monoculture on mustard (*Sinapis alba* ‘Albatros’) roots growing on Knop medium (0.2% wt/vol). The hatching of the juveniles was stimulated by adding 3 mM ZnCl_2_. On three consecutive washes with sterile water, 60–70 *H*. *schachtii* second‐stage juveniles (J2s) were inoculated onto the Knop medium plate containing 12‐day‐old *Arabidopsis* plants under sterile conditions. Two plants were used in one Petri dish and experiments were repeated at least three times independently, with 20–30 plants per genotype in each replicate. The numbers of female nematodes per plant were counted using a stereomicroscope (Leica Microsystems) at 14 dpi. Subsequently, the infection rate per centimetre of root length was determined after scanning roots with the WinRhizo root image analysis system. The female nematodes and female‐associated syncytia were outlined, and the area was calculated at 14 dpi using an M165C stereomicroscope equipped with LAS v. 4.3 image analysis software (Leica Microsystems). At 42 dpi, cysts were randomly selected and crushed in between slides. The contents were then transferred into a counting dish and the number of eggs/J2s were counted using an S4E stereomicroscope (Leica Microsystems). Cyst sizes were measured at 42 dpi.

### 4.4 Nematode attraction assay

Nematode attraction assays were conducted on all glutathione‐deficient mutants, according to Dalzell et al. (2011), with some modifications. Uniform circular counting wells 6 mm in diameter, attached through cylindrical tunnels (2.5 mm depth × 20 mm length), were constructed in a 2% (wt/vol) water agar plate. The cylindrical channels were created by placing a 20‐mm long plastic tube constructed from the handle of a regular inoculation loop onto the agar surface immediately after pouring. With the aid of fine forceps, the tubular plastic was removed once the medium had solidified. The wells were cut with a small transfer glass pipette at either side of the central channels to create a 6‐mm diameter well. Agar plugs were excised from the plate close to the roots carrying root exudates from Col‐0 and mutant plants raised on Knop medium and subsequently transferred into the counting wells. Around 60–80 J2s were put in the centre of the cylindrical linking channel and kept at room temperature in a dark place. Over a 2‐h period, the number of nematodes that moved to either one or the other well were counted and considered as attracted by the exudates of the respective agar plug. Experiments were repeated three times independently with six plates each (*n* = 18). The attraction rate (%) was calculated from the total number of nematodes applied.

### 4.5 Biochemical analysis

For biochemical analysis, 12‐day‐old *Arabidopsis* plants were inoculated with *H*. *schachtii*. Tiny root segments containing infection sites around the nematode at 10 hpi and female‐associated syncytia at 10 dpi were dissected under a stereomicroscope and collected in liquid nitrogen. The root tip or lateral root primordia were excluded during collection. Similarly, the control sample was collected from the corresponding root segments of uninfected plants. Cysteine and glutathione in plant tissues were extracted and quantified by HPLC, according to Anoman et al. (2019). Similarly, samples were collected at 10 hpi and camalexin was measured as described previously (Koprivova et al., 2019).

### 4.6 RT‐qPCR

To collect root tissues for gene expression analysis, *Arabidopsis* Col‐0 plants were raised and inoculated using 4‐day‐old J2s of *H*. *schachtii*, as described earlier. Several hundred tiny root pieces (c.0.2 cm) containing infection sites around the nematodes at 10 hpi were dissected under a stereomicroscope and collected in liquid nitrogen. Total RNA was extracted from the frozen root tissues using an RNeasy Plant Mini kit (Qiagen) as per the manufacturer's instructions. The RNA concentration was checked with a NanoDrop (Thermo Fisher Scientific), and reverse transcription was performed using a High Capacity cDNA Reverse Transcription Kit (Life Technologies), as per the manufacturer's instructions. The quantitative PCRs were carried out in a volume of 20 μl comprising 10 μl of Fast SYBR Green qPCR Master Mix with uracil‐DNA, 6‐carboxy‐x‐rhodamine, and glycosylase (Invitrogen), 0.5 μl of forward primer, 0.5 μl of reverse primer (10 μM), water, and 1 μl of cDNA (c.100 ng), respectively. The qPCR analysis was conducted using a StepOne Plus Real‐Time PCR (Applied Biosystems) system based on a two‐step amplification protocol, with the following cycling conditions: 95°C for 3 min followed by 40 cycles of 95°C for 10 s and 60°C for 30 s. For each sample run, a melt‐curve analysis was done following 95°C for 15 s, 65–95°C with 0.5°C incremental progress yielding a single peak. As a negative control, a water‐containing nontemplate reaction was included. The transcript abundance of targeted genes was computed from three biological replicates per treatment, with three technical replicates for each biological sample. Relative expression was calculated by normalizing target gene expression to the abundance of the *Arabidopsis* housekeeping genes *18S rRNA* and *UBQ10* (Pfaffl, 2001). The gene‐specific primer pairs used for RT‐qPCR are provided in Table S2.

### 4.7 Confocal laser scanning microscopy imaging and ratiometric analysis

Confocal laser scanning microscopy (CLSM) imaging and ratiometric analyses in Col‐0, *rax1*, *pad2*, and *cad2* root tissues expressing Grx1‐roGFP2 were conducted according to Schwarzländer et al. (2008). Plants were grown in Knop medium (0.2% wt/vol) in 35‐mm Petri dishes with a 14‐mm glass microwell (Mattek) and 10‐day‐old seedlings were inoculated with 50 J2s of CNs. While we were able to grow and inoculate all *gsh1* alleles, *zir1* showed extremely poor growth under these conditions. We therefore excluded *zir1* from roGFP experiments.

The ratio of fluorescence intensity after excitation at 405 and 488 nm of the glutathione redox potential sensor Grx1‐roGFP2 was examined in uninfected root tissues and nematode infection sites at 10 hpi. The dynamic range of the sensor (405/488 nm ratio) was determined by in situ calibration (0.5–2.0) and was set as the minimum/maximum value of the ratio false colour scale. For calibration, 5 mM DPS solution as oxidizing and 10 mM DTT solution as reducing agents were used. Images were captured using a confocal microscope (LSM 780; Zeiss) with a 25× lens using the multitrack mode, with line switching and averaging two frames. The fluorescence of Grx1‐roGFP2 was collected at 505–530 nm. RRA v. 1.2 software (Fricker, 2016) was used for ratiometric image analysis. To minimize impact by cell wall autofluorescence, values were measured on regions of interest (ROIs) in nuclei of cells near infection sites. Values outside the minimum/maximum value of the ratio (0.5–2.0) were removed. Statistical analysis was performed on log‐transformed values.

### 4.8 Statistical analysis

All data were analysed using the statistical software GraphPad Prism v. 8.4.3 for Windows. Infection assays were carried out with 20–30 plants for each genotype and replicated at least three times independently. The normality of data was assessed using the Kolmogorov–Smirnov test/Shapiro–Wilk test (α < 0.05). The specific statistical method used for the data set of each experiment is described in the figure legends. Data are presented as mean ± *SE*, and corresponding *p* values are indicated either in the figure or in the figure legends.

## Supporting information


**FIGURE S1** Expression profile of *GSH1* and *GSH2* in RNA‐Seq data (Siddique et al., 2021)

**FIGURE S2** Nematode attraction assays towards root exudates of glutathione‐deficient mutant plants

**FIGURE S3** Reduced glutathione levels do not impair basal defence responses in uninfected roots

**FIGURE S4** Genotyping results of glutathione deficient mutants (*rax1*, *pad2*, *cad2*, and *zir1*) used in this study

**TABLE S1** Overview of glutathione biosynthesis gene *GSH1* and *GSH2* expression patterns in *Arabidopsis* roots at migratory and sedentary stage of *Heterodera schachtii* infection in published transcriptomic data

**TABLE S2** List of primers used for validation of the mutant lines tested in this study

## Data Availability

The data that support the findings of this study are available from the corresponding author upon reasonable request.
