# Supplementary material for: Glutathione contributes to plant defence against parasitic cyst nematodes
Source: Mol Plant Pathol. 2022 Mar 29;23(7):1048–59. doi: 10.1111/mpp.13210 (PMC9190975; doi:10.1111/mpp.13210)
Supplement: Supplementary file 1 — FIGURE S1 Expression profile of GSH1 and GSH2 in RNA‐Seq data (Siddique et al., 2021) [file MPP-23-1048-s001.docx]

**Fig S1: Expression profile of GSH1 and GSH2 in RNA-Seq data (Siddique et al., 2021).** hpi, hours post inoculation; dpi, days post inoculation,

**
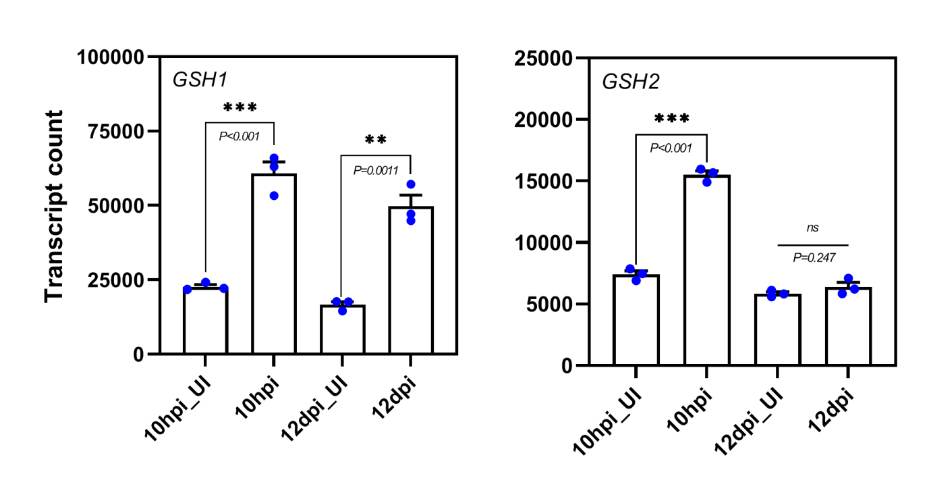
**
