# Supplementary material for: Glutathione contributes to plant defence against parasitic cyst nematodes
Source: Mol Plant Pathol. 2022 Mar 29;23(7):1048–59. doi: 10.1111/mpp.13210 (PMC9190975; doi:10.1111/mpp.13210)

**Fig. S2. Nematode attraction assays towards root exudates of glutathione-deficient mutant plants.** Nematode attractiveness to root exudates of *GSH1* mutants compared with Col-0 plants (control = control agar). Experiments were repeated three times independently for each mutant with six plates each (*n* = 18). The attraction rate (%) was calculated from the total number of applied nematodes. Bars represent mean ± SE. Data were analyzed using two-tailed Student’s t‐test (P < 0.05). Asterisks indicate significant differences (*P* < 0.05).


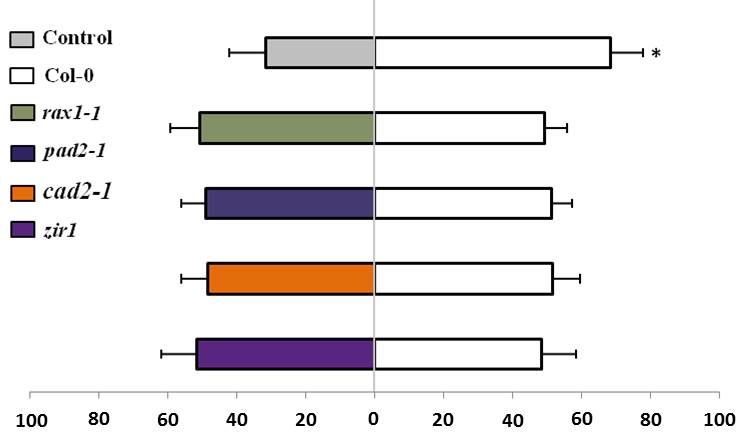

Supplement: Supplementary file 2 — FIGURE S2 Nematode attraction assays towards root exudates of glutathione‐deficient mutant plants [file MPP-23-1048-s006.docx]
