# Supplementary material for: Glutathione contributes to plant defence against parasitic cyst nematodes
Source: Mol Plant Pathol. 2022 Mar 29;23(7):1048–59. doi: 10.1111/mpp.13210 (PMC9190975; doi:10.1111/mpp.13210)
Supplement: Supplementary file 3 — FIGURE S3 Reduced glutathione levels do not impair basal defence responses in uninfected roots [file MPP-23-1048-s004.docx]

**Fig. S3. Reduced glutathione levels do not impair basal defense responses in uninfected roots.** Expression of defense marker genes in uninfected roots of GSH-deficient mutants.

**
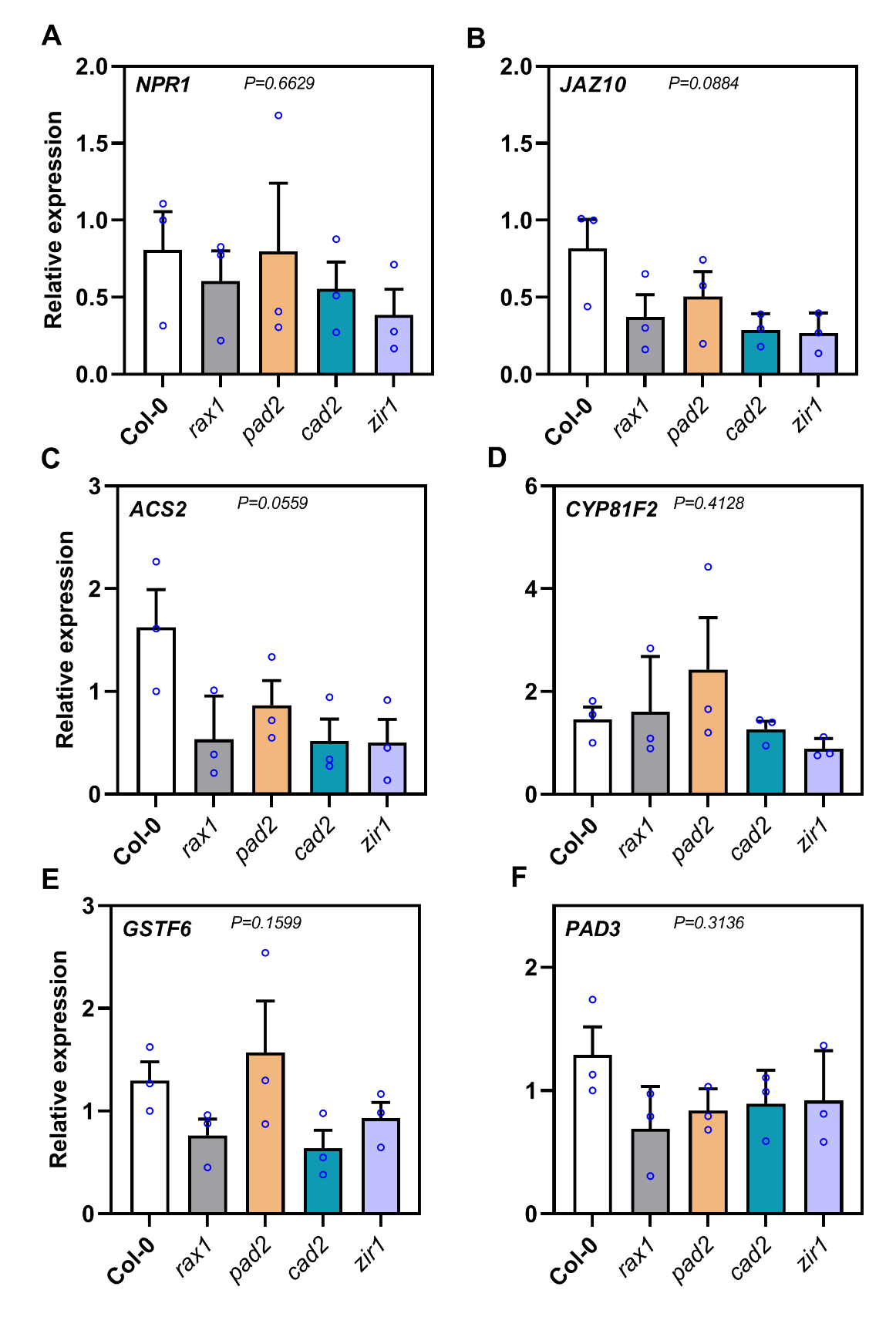
**
