# Supplementary material for: Glutathione contributes to plant defence against parasitic cyst nematodes
Source: Mol Plant Pathol. 2022 Mar 29;23(7):1048–59. doi: 10.1111/mpp.13210 (PMC9190975; doi:10.1111/mpp.13210)

**Fig. S4. Genotyping results of glutathione deficient mutants (*rax1*, *pad2, cad2* and *zir1*) used in this study.** The GSH1 mutants, *rax1*, *pad2,* and *zir1* have single point mutations leading to changes in amino acid (AA) synthesis, and the line *cad2* has a 6 bp long deletion. The point or deletion mutations were identified (shown in a red box) via PCR using primers listed in **Supplementary Table S2** and subsequently, the single point mutation/deletion was verified by sequencing. The shown sequences are only for the PCR products amplified by the respective primers.


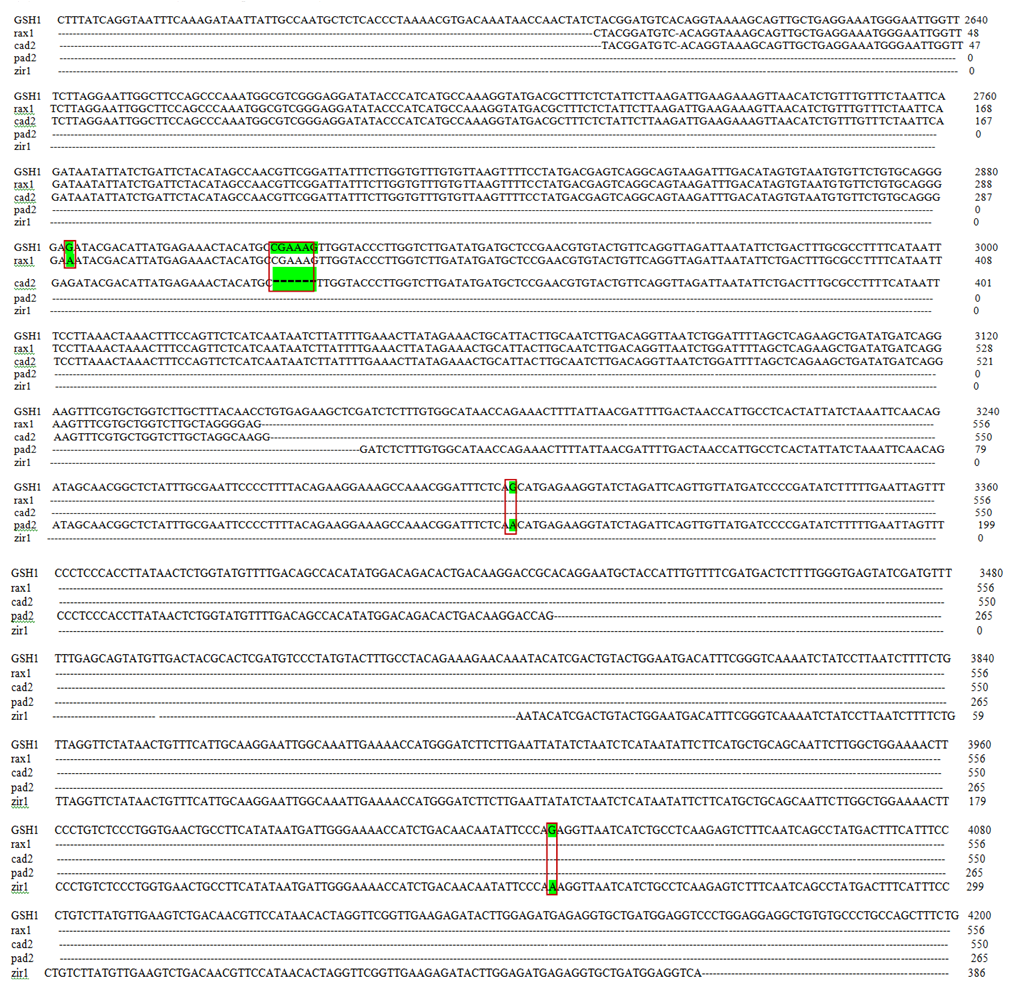

Supplement: Supplementary file 4 — FIGURE S4 Genotyping results of glutathione deficient mutants (rax1, pad2, cad2, and zir1) used in this study [file MPP-23-1048-s003.docx]
