# Supplementary material for: Glutathione contributes to plant defence against parasitic cyst nematodes
Source: Mol Plant Pathol. 2022 Mar 29;23(7):1048–59. doi: 10.1111/mpp.13210 (PMC9190975; doi:10.1111/mpp.13210)
Supplement: Supplementary file 5 — TABLE S1 Overview of glutathione biosynthesis gene GSH1 and GSH2 expression patterns in Arabidopsis roots at migratory and sedentary stage of Heterodera schachtii infection in published transcriptomic data [file MPP-23-1048-s005.docx]

**Table S1. Overview of glutathione biosynthesis gene *GSH1* and *GSH2* expression patterns in Arabidopsis roots at migratory and sedentary stage of *H*. *schachtii* infection in published transcriptomic data.**

| **Gene** | **Locus** | **Fold change compared with uninfected control** | |
| --- | --- | --- | --- |
|  |  | **Migratory (10 hpi)** | **Sedentary (5+15 dpi)** |
| *GSH1* | At4g23100 | 2.1* | 6.5* |
| *GSH2* | At5g27380 | 2.1* | 0.8 |

For the migratory stage, root sections containing infection sites at 10 hours post infection (hpi) were analyzed and compared with uninfected control roots (Mendy et al., 2017). For the sedentary stage, microaspirated syncytia at 5 and 15 days post-infection (dpi) were pooled and compared with control roots (Szakasits et al., 2009). Asterisks indicate significant difference to control.
