# Supplementary material for: Glutathione contributes to plant defence against parasitic cyst nematodes
Source: Mol Plant Pathol. 2022 Mar 29;23(7):1048–59. doi: 10.1111/mpp.13210 (PMC9190975; doi:10.1111/mpp.13210)
Supplement: Supplementary file 6 — TABLE S2 List of primers used for validation of the mutant lines tested in this study [file MPP-23-1048-s002.docx]

**Table S2. List of primers used in this study.**

| **Gene/mutant allele** | **Locus** | **Forward primer** | **Reverse primer** |
| --- | --- | --- | --- |
| *rax1* | AT4G23100 | TGCTCTCACCCTAAAACGTG | AGCAAGACCAGCACGAAACT |
| *pad2* | AT4G23100 | TCGTGCTGGTCTTGCTTTAC | CGGTCCTTGTCAGTGTCTGT |
| *cad2* | AT4G23100 | TGCTCTCACCCTAAAACGTG | AGCAAGACCAGCACGAAACT |
| *zir1* | AT4G23100 | ACGCACTCGATGTCCCTATG | GACCTCCATCAGCACCTCTC |
| 18S | 18S RRNA | GGTGGTAACGGGTGACGGAGAAT | CGCCGACCGAAGGGACAAGCCGA |
| *NPR1* | AT1G64280 | GAGTTGCACTTGCTCAACGTC | GCTATCTTTACACCCGGTGATG |
| *JAZ10* | AT5G13220 | TCGCAAGGAGAAAGTCACTGCAAC | CGATTTAGCAACGACGAAGAAGGC |
| *ACS2* | AT1G01480 | GGATGGTTTAGGATTTGCTTTG | GCACTCTTGTTCTGGATTACCTG |
| *CYP81F2* | AT5G57220 | ATCGTGCTAGTGAACGCTTG | TTCGTCCGTTACCAAACACC |
| *PAD3* | AT3G26830 | GGGTACCATACTTGTTGAGATGG | TTGATGATCTCTTTGGCTTCC |
| *GSH1* | AT4G23100 | CCAGCTTTCTGGGTGGGTTT | GCTTCCTTGTAGCCTCTGCG |
| *GSH2* | AT5G27380 | TGGCTAAAGCTTGGTTGGAGT | AACCACTGCGACTGCTTGG |
| UBQ10 | AT4G05320 | GGCCTTGTATAATCCCTGATGAATAAG | AAAGAGATAACAGGAACGGAAACATAG |
